# Study on Interfacial Crack of Piezoelectric Bimaterials Under Dynamic Loading

**DOI:** 10.3390/ma19050964

**Published:** 2026-03-02

**Authors:** Yani Zhang, Junlin Li, Xiangyu Li, Junye Ma

**Affiliations:** 1 School of Applied Science, Taiyuan University of Science and Technology, Taiyuan 030024, China; 2 School of Materials Science and Engineering, Taiyuan University of Science and Technology, Taiyuan 030024, China

**Keywords:** integral transformation, piezoelectric bimaterials, dynamic fracture, interfacial crack, non-dimensional function

## Abstract

To meet the requirements of effectiveness and strength in actual engineering, based on the dynamic fracture characteristics, the dynamic propagation of orthogonal anisotropic interface cracks in piezoelectric bimaterials was analyzed. By performing Laplace transformation and Fourier transformation on the governing equations, the problem was transformed into a singular integral equation. Using the Chebyshev point method and Laplace inversion, the stress and electric displacement intensity factors at the crack tip of the orthogonal anisotropic interface were obtained. The results show that the crack length affects the dimensionless function. The longer the crack, the larger the dimensionless function. Under certain conditions, the smaller the elastic parameters, the smaller the dimensionless dynamic stress intensity factor. At the same time, the impact time also affects the dynamic crack propagation. With the passage of time, the dimensionless function first increases, then reaches a peak, and finally oscillates and converges to the static value. On this basis, the response surface method was used for analysis and prediction. The R^2^ value of the random forest model is 0.9886, which indicates that the model has high predictive accuracy. When the optimal values of A ($d_{1}/a$), B ($c_{p}t/a$) and C ($c_{44}^{\left(2\right)}/c_{44}^{\left(1\right)}$) are 0.4045, 1.6797 and 1.9035 respectively, the stress intensity reaches its maximum value of 1.3375.

## 1. Introduction

Piezoelectric materials have been widely used in advanced equipment, instruments and materials due to their electromechanical coupling properties. However, during manufacturing or usage, defects such as cracks are inevitable, which may lead to structural damage or device failure. Therefore, the crack problem of piezoelectric materials has attracted considerable attention [1,2,3,4,5]. In recent years, piezoelectric bimaterials have been widely applied in devices. The sudden change in materials on both sides of the interface may cause stress concentration, which is one of the important reasons for material failure [6,7,8]. Li et al. [9] analyzed the SH-wave scattering by interfacial cracks in piezoelectric ceramic-polymer composites. Their core objective was to establish a dynamic response analysis framework for cracks under a steady-state wave field, and they preliminarily investigated the effects of frequency, incident angle, and material properties on crack propagation. This work provides a fundamental wave-based analysis model for subsequent dynamic fracture research; however, it does not address the transient response under impact loading or the coupling behavior in complex anisotropic material systems.

Research on the behavior of interface crack propagation has achieved fruitful results. Loboda et al. conducted studies on the conductive interface cracks between one-dimensional hexagonal piezoelectric quasicrystals and ordinary piezoelectric materials, analyzing the coupling effect between the cracks and the far-field electrodes, and clarifying the energy-evolution law of piezoelectric hetero-interface cracks, providing theoretical references for the analysis of related issues [10,11,12]. Sun et al. [13] proposed the arbitrary-order generalized finite difference method (GFDM), providing a new numerical solution path for homogeneous and dual-material piezoelectric crack problems. The crack propagation behavior of multiple interfaces caused by circular holes under dynamic loading was studied, and the influence law of functional gradient characteristics on the dynamic response of cracks was clarified, confirming that the gradient distribution of material parameters would significantly change the stress-electric field concentration effect at the crack tip [14]. Based on the improved mechanical energy release rate criterion, the dynamic expansion characteristics of cracks in piezoelectric plates were revealed [15]. In terms of innovative analysis methods, a new interaction integral method was proposed, achieving precise analysis of dynamic impermeable interface cracks in heterogeneous piezoelectric materials, effectively improving the accuracy of field quantity calculation at the crack tip [16]. Liu et al. [17,18,19] based on the non-local piezoelectric theory, systematically studied the dynamic response of I-type penetrating cracks in piezoelectric materials, derived the dynamic analytical solutions for finite-penetrating and complete-penetrating cracks, and improved the theoretical system of dynamic fracture in piezoelectric materials. Afshar et al. [20] studied the dynamic fracture behavior of multiple intruded cracks in functional gradient piezoelectric strips [20]. Wünsche et al. [21] used the symmetrical Galerkin boundary element method to establish an analysis model for dynamic cracks in piezoelectric bodies under harmonic loading, providing an efficient solution method for piezoelectric fracture problems under harmonic loading. Yu et al. [22] based on the extended finite element method (XFEM), established a numerical solution framework for the dynamic crack of heterogeneous piezoelectric materials under force–electric coupling loading, providing an efficient calculation tool for interface fracture problems in complex configurations.

Through in-depth analysis of the existing research, we can find that the electromechanical coupling constitutive relationship of orthotropic anisotropic materials is more complex, and the direction dependence of their physical parameters leads to the inability to directly transplant the existing theoretical models for isotropic or functionally graded materials; at the same time, although some studies involve dynamic loads, they are mainly concentrated on harmonic loads or do not fully capture the transient response characteristics under impact loads; and the existing research mostly focuses on specific types of conductive cracks, non-conductive cracks, etc., and mainly focuses on the analysis of the influence of a single factor, without deeply exploring the coupling effect and quantitative influence laws of multiple parameters such as elastic parameters, crack length, and impact time, which is difficult to meet the systematic research requirements of this article on the dynamic expansion mechanism of interface cracks in orthotropic anisotropic piezoelectric dual materials.

In summary, although the existing research has laid a foundation for the crack problem of dual-layer materials, it has obvious limitations in adapting to the specific research object, load conditions, and analysis goals of this article.

The above research has constructed a research framework for the crack problem of piezoelectric materials from aspects such as material system, crack form, analysis method, and load type. However, the theory and methods still have obvious limitations in applying to the orthogonal anisotropic piezoelectric dual-material interface crack problem under dynamic loads. On one hand, the existing research on dual-material interface cracks mostly focuses on static/sinusoidal loads, isotropic/functional gradient piezoelectric media, or do not consider the orthogonal anisotropy characteristics of the materials. The electromechanical coupling constitutive relationship of orthogonal anisotropic piezoelectric dual-material is more complex, and the transient response laws under dynamic impact loads are significantly different from those of conventional media. The existing static theories and analysis methods under sinusoidal loads cannot be directly transplanted. On the other hand, most dynamic-crack research focuses on internal cracks of a single piezoelectric material or does not deeply explore the coupling effects of multiple parameters and quantitative influence rules. There is no complete analytical–numerical coupled solution system for the dynamic-fracture problem of orthogonal anisotropic piezoelectric dual-material interfaces, and the research on the quantitative influence of key parameters such as elastic parameters, crack length, and impact time and the interaction between parameters is still scarce. In addition, most existing studies adopt a single analytical or numerical method for crack analysis. The research of integrating the response-surface method into dynamic fracture analysis to achieve rapid prediction of multiple parameters and identification of optimal working conditions is still in the exploratory stage.

During manufacturing and usage, cracks may occur at the interface due to manufacturing level and other factors. Meanwhile, under dynamic loads, the material thickness, elastic constants, etc., of the piezoelectric bimaterials will affect the dynamic propagation of interface cracks, which may lead to device failure and losses. However, there are few articles on this topic. Moreover, the dynamic propagation of interface cracks in piezoelectric bimaterials has limitations in software simulation and experiments. Therefore, precise quantitative analysis of the dynamic fracture problem of interface cracks in piezoelectric bimaterials is of great significance for structural design and safety.

The motivation for this study stems from the urgent need to address the dynamic fracture behavior of interface cracks in piezoelectric bimaterials. This is a topic that has received limited attention in existing literature, especially concerning the complex scenario involving orthogonally anisotropic materials under dynamic loads. Piezoelectric materials play a critical role in advanced equipment, instruments, and sensors due to their unique electromechanical coupling properties. However, their brittle nature and inevitable defects, such as cracks, during manufacturing and use can lead to structural damage or even device failure. While research on static fracture problems has a certain foundation, many piezoelectric components in practical applications are often subjected to dynamic loads, making the study of dynamic fracture behavior particularly important and challenging. Currently, precise quantitative analysis of dynamic interface cracks in orthogonally anisotropic piezoelectric bimaterials, whether in theoretical analysis, numerical simulation, or experimental verification, faces significant deficiencies.

Our primary contribution lies in the innovative application and integration of advanced analytical and computational methods to deeply explore this complex problem. Specifically, we employed the powerful mathematical tools of Laplace transformation and Fourier transformation, successfully converting the governing equations that describe the dynamic expansion of orthogonally anisotropic interface cracks into a complex singular integral equation. Subsequently, we applied a high-precision Chebyshev point method for numerical discretization of this integral equation, combined with efficient Laplace inversion techniques, to accurately solve for the stress-intensity factors and electric-displacement-intensity factors at the crack tip. This combined analytical and numerical approach provides a solid foundation for understanding dynamic-fracture mechanisms. Furthermore, to further enhance analysis efficiency and predictive capability, we proactively integrated the Response Surface Methodology (RSM) and specifically developed and applied a high-precision Random Forest (Random Forest) machine learning model (with a determination coefficient R^2^ as high as 0.9886, demonstrating the model’s excellent goodness of fit and predictive accuracy) for effective analysis and prediction of complex multi-parameter influences.

This multi-level comprehensive method offers significant added value. It not only precisely reveals the quantitative influence laws of key factors such as crack length, elastic parameters (e.g., modulus), and impact time on dynamic fracture behavior—for example, revealing the positive correlation between crack length and the dimensionless function, as well as the inverse relationship between elastic parameters and the dimensionless dynamic stress intensity factor under certain conditions—but also captures the dynamic evolution process induced by impact time, where the dynamic crack response first increases, reaches a peak, and then oscillates and converges to the static value. Moreover, it ultimately enables the identification of optimal conditions affecting stress intensity. This in-depth and quantitative understanding greatly enhances the prediction accuracy of dynamic crack propagation mechanisms, providing a key theoretical basis for evaluating and optimizing the dynamic reliability of piezoelectric devices, thereby significantly improving the operational reliability and structural safety of piezoelectric equipment in practical engineering applications. By directly addressing the limitations of current software simulations in handling dynamic fracture problems of complex anisotropic materials, as well as the challenges of experimental research in terms of cost, scale, and dynamic loading control, this study aims to fill a critical gap in the field and provide important scientific support and technical guidance for the future design of more robust, safer, and more reliable piezoelectric smart structures.

## 2. Establishment of the Model

**Figure 1:**
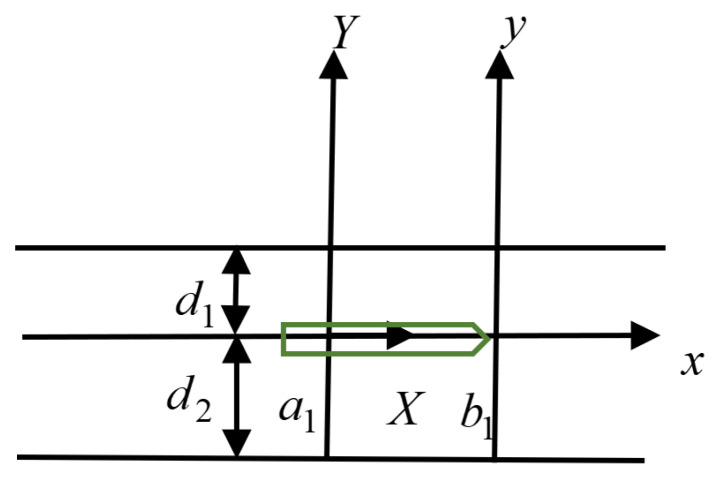
Structure of the piezoelectric bimaterials model with cracks (The green part in the picture indicates the direction of the crack movement).

As shown in Figure 1, the thicknesses of the two materials are $d_{1}$ and $d_{2}$, respectively. The crack is from $a_{1}$ to $b_{1}$. With the interface direction as the X-axis and the thickness direction as the Y-axis. Let us assume that the polarization direction of the piezoelectric dual materials is the Z-axis, the structure is orthotropic, and the symmetry axes of the materials are perfectly aligned at the interface.

The governing equation for the crack in the piezoelectric bimaterial orthotropic interface is:

(1) $$c_{55}^{\left(k\right)}\frac{\partial^{2}w}{\partial X^{2}}+c_{44}^{\left(k\right)}\frac{\partial^{2}w}{\partial Y^{2}}+e_{15}^{\left(k\right)}\frac{\partial^{2}\phi }{\partial X^{2}}+e_{24}^{\left(k\right)}\frac{\partial^{2}\phi }{\partial Y^{2}}=\rho \frac{\partial^{2}w}{\partial t^{2}}$$

(2) $$e_{15}^{\left(k\right)}\frac{\partial^{2}w}{\partial X^{2}}+e_{24}^{\left(k\right)}\frac{\partial^{2}w}{\partial Y^{2}}-\varepsilon_{11}^{\left(k\right)}\frac{\partial^{2}\phi }{\partial X^{2}}-\varepsilon_{22}^{\left(k\right)}\frac{\partial^{2}\phi }{\partial Y^{2}}=0$$

In the above equations, w=w(x,y,t) denotes the mechanical displacements, φ(x,y,t) represents the electric potentials, ρ is the mass density, $c_{55}^{\left(k\right)}$ and $c_{44}^{\left(k\right)}$ are the elastic stiffness constants, $e_{15}^{\left(k\right)}$ and $e_{24}^{\left(k\right)}$ stand for the piezoelectric constants, and $\varepsilon_{11}^{\left(k\right)}$ and $\varepsilon_{22}^{\left(k\right)}$ are the dielectric constants. Here, k=1,2 correspond to piezoelectric material 1 and piezoelectric material 2, respectively.

Introduce the transformation x=X−vt, y=Y, then Equations (1) and (2) becomes:

(3) $$\left[\begin{array}{cccc} \frac{c_{55}^{\left(k\right)}}{c_{44}^{\left(k\right)}}-\frac{v^{2}}{{\bar{c_{k}}}^{2}} & 1 & \frac{e_{15}^{\left(k\right)}}{c_{44}^{\left(k\right)}} & \frac{e_{24}^{\left(k\right)}}{c_{44}^{\left(k\right)}}\\ \frac{e_{15}^{\left(k\right)}}{c_{44}^{\left(k\right)}} & \frac{e_{24}^{\left(k\right)}}{c_{44}^{\left(k\right)}} & -\frac{\varepsilon_{11}^{\left(k\right)}}{c_{44}^{\left(k\right)}} & -\frac{\varepsilon_{22}^{\left(k\right)}}{c_{44}^{\left(k\right)}} \end{array}\right]\left[\begin{array}{c} \frac{\partial^{2}w}{\partial x^{2}}\\ \frac{\partial^{2}w}{\partial y^{2}}\\ \frac{\partial^{2}\phi }{\partial x^{2}}\\ \frac{\partial^{2}\phi }{\partial y^{2}} \end{array}\right]=\left[\begin{array}{c} \Psi^{\left(k\right)}\\ 0 \end{array}\right]$$

Perform the Laplace transform on time t and the Fourier transform on x. The solution of Equation (3) can be expressed as

(4) $$[\begin{array}{c} w^{*}\left(x,y,p\right)\\ \phi^{*}\left(x,y,p\right) \end{array}]=\frac{1}{2\pi }\int_{-\infty }^{+\infty }\left[\begin{array}{cc} \chi_{11}^{\left(k\right)} & \chi_{12}^{\left(k\right)}\\ \chi_{21}^{\left(k\right)} & \chi_{22}^{\left(k\right)} \end{array}\right][\begin{array}{c} A_{11}^{\left(k\right)}e^{(3-2k)s\gamma_{1}^{\left(k\right)}y}\\ A_{12}^{\left(k\right)}e^{(3-2k)s\gamma_{2}^{\left(k\right)}y} \end{array}]e^{-isx}ds$$

where

$$\chi_{1\eta }^{\left(k\right)}=\frac{e_{24}^{\left(k\right)}}{c_{44}^{\left(k\right)}}\gamma_{\eta }^{(k{)}^{2}}-s^{2}\frac{e_{15}^{\left(k\right)}}{c_{44}^{\left(k\right)}},\;\chi_{2\eta }^{\left(k\right)}=\gamma_{\eta }^{(k{)}^{2}}-s^{2}[(\frac{c_{55}^{\left(k\right)}}{c_{44}^{\left(k\right)}}-\frac{v^{2}}{{\bar{c_{k}}}^{2}})+\frac{2vpi}{{\bar{c_{k}}}^{2}s}+\frac{p^{2}}{{\bar{c_{k}}}^{2}s^{2}}],\;\eta =1,2.$$

The value of $\gamma_{\eta }^{\left(k\right)}$ can be found in Appendix A.

By analyzing the model, it can be concluded that the boundary conditions that the cracks must satisfy are

(5) $$\sigma_{yz}(x,d_{1},t)=\sigma_{yz}(x,-d_{2},t)=0$$

(6) $$w(x,0^{+},t)=w(x,0^{-},t),\;D_{y}(x,0^{+},t)=D_{y}(x,0^{-},t)$$

(7) $$\begin{array}{l} \sigma_{yz}(x,0^{+},t)=\sigma_{yz}(x,0^{-},t)=-\tau_{0}H(t)\\ D_{y}(x,0^{+},t)=D_{y}(x,0^{-},t)=-D_{0}H(t) \end{array}$$

where $H(t)=\left\{\begin{array}{c} 0,\begin{array}{c} t<0 \end{array}\\ 1,\begin{array}{c} t>0 \end{array} \end{array}\right.$.

By analyzing the model, it can be concluded that the boundary conditions that the cracks must satisfy are

(8) $$\sigma_{yz}^{*}(x,d_{1},p)=\sigma_{yz}^{*}(x,-d_{2},p)=0$$

(9) $$w^{*}(x,0^{+},p)=w^{*}(x,0^{-},p),\;D_{y}^{*}(x,0^{+},p)=D_{y}^{*}(x,0^{-},p)$$

(10) $$\sigma_{yz}^{*}(x,0^{+},p)=\sigma_{yz}^{*}(x,0^{-},p)=-\frac{\tau_{0}}{p},\;D_{y}^{*}(x,0^{+},p)=D_{y}^{*}(x,0^{-},p)=-\frac{D_{0}}{p}$$

The electric displacement corresponding to the stress in Equation (4) is

(11) $$\left[\begin{array}{c} \sigma_{yz}^{*}\\ D_{y}^{*} \end{array}\right]=\frac{1}{2\pi }\int_{-\infty }^{+\infty }\left(\left[\begin{array}{cc} c_{44}^{\left(k\right)} & e_{24}^{\left(k\right)}\\ e_{15}^{\left(k\right)} & -\varepsilon_{22}^{\left(k\right)} \end{array}\right]\left[\begin{array}{cc} \chi_{11}^{\left(k\right)} & \chi_{12}^{\left(k\right)}\\ \chi_{21}^{\left(k\right)} & \chi_{22}^{\left(k\right)} \end{array}\right]\left[\begin{array}{c} \gamma_{1}^{\left(k\right)}e^{(3-2k)s\gamma_{1}^{\left(k\right)}y}A_{11}^{\left(k\right)}\\ \gamma_{2}^{\left(k\right)}e^{(3-2k)s\gamma_{2}^{\left(k\right)}y}A_{12}^{\left(k\right)} \end{array}\right](3-2k)s\right)e^{-isx}ds$$

## 3. Solution of the Model

In the process of crack propagation, the expansion of the crack is affected by a variety of complex factors. To more accurately describe the crack propagation behavior, introducing a density function is an effective method. Therefore, the following density function is introduced:

(12) $$h_{w}(x)=\frac{d}{dx}[w(x,0^{+},p)-w(x,0^{-},p)]$$

(13) $$h_{\phi }(x)=\frac{d}{dx}[\phi (x,0^{+},p)-\phi (x,0^{-},p)]$$

Since displacement and potential are continuous, we can obtain:

(14) $$\int_{a_{1}}^{b_{1}}h_{w}(x)dx=0,\;x\notin (a_{1},b_{1})$$

(15) $$\int_{a_{1}}^{b_{1}}h_{\phi }(x)dx=0,\;x\notin (a_{1},b_{1})$$

Based on the boundary conditions (8–10) and Equations (12) and (13), the following conclusion can be obtained:

(16) $$\left[\begin{array}{cc} \chi_{11}^{\left(1\right)} & \chi_{12}^{\left(1\right)}\\ \chi_{21}^{\left(1\right)} & \chi_{22}^{\left(1\right)} \end{array}\right]\left[\begin{array}{c} A_{11}\\ B_{11} \end{array}\right]-\left[\begin{array}{cc} \chi_{11}^{\left(2\right)} & \chi_{12}^{\left(2\right)}\\ \chi_{21}^{\left(2\right)} & \chi_{22}^{\left(2\right)} \end{array}\right]\left[\begin{array}{c} A_{12}\\ B_{12} \end{array}\right]=\frac{i}{s}\int_{a_{1}}^{b_{1}}\left[\begin{array}{c} h_{w}(s)\\ h_{\phi }(s) \end{array}\right]e^{isf}df=\left[\begin{array}{c} H_{1}\\ H_{2} \end{array}\right]$$

(17) $$\left[\begin{array}{c} A_{11}^{\left(1\right)}\\ A_{12}^{\left(1\right)}\\ A_{11}^{\left(2\right)}\\ A_{12}^{\left(2\right)} \end{array}\right]=\frac{1}{A^{*}}\left[\begin{array}{cc} A_{1} & B_{1}\\ A_{2} & A_{2}\\ A_{1}^{*} & B_{1}^{*}\\ A_{2}^{*} & B_{2}^{*} \end{array}\right]\left[\begin{array}{c} H_{1}\\ H_{2} \end{array}\right]$$

The values of $A_{1},B_{1},A_{2},B_{2},A_{1}^{*},B_{1}^{*},A_{2}^{*},B_{2}^{*},A^{*}$ can be found in Appendix A.

Substituting Equation (17) into Equation (11) yields

(18) $$\left[\begin{array}{c} \sigma_{yz}^{*}(x,0^{+},p)\\ D_{y}^{*}(x,0^{+},p) \end{array}\right]=\underset{y\to 0^{+}}{\lim}\frac{1}{2\pi }\int_{-\infty }^{+\infty }\frac{se^{s\gamma y}}{A^{*}}\left[\begin{array}{cc} C_{1} & C_{2}\\ C_{3} & C_{4} \end{array}\right]\left[\begin{array}{c} H_{1}\\ H_{2} \end{array}\right]e^{-isx}ds$$

where

$$C_{1}=\sum_{i=1}^{2}\left(c_{44}^{\left(1\right)}\chi_{1i}^{\left(1\right)}+e_{24}^{\left(1\right)}\chi_{2i}^{\left(1\right)}\right)A_{i}\gamma_{i}^{\left(1\right)},$$

$$C_{2}=\sum_{i=1}^{2}\left(c_{44}^{\left(1\right)}\chi_{1i}^{\left(1\right)}+e_{24}^{\left(1\right)}\chi_{2i}^{\left(1\right)}\right)B_{i}\gamma_{i}^{\left(1\right)},$$

$$C_{3}=\sum_{i=1}^{2}\left(e_{15}^{\left(1\right)}\chi_{1i}^{\left(1\right)}-\varepsilon_{22}^{\left(1\right)}\chi_{2i}^{\left(1\right)}\right)A_{i}^{*}\gamma_{i}^{\left(1\right)},$$

$$C_{4}=\sum_{i=1}^{2}\left(e_{15}^{\left(1\right)}\chi_{1i}^{\left(1\right)}-\varepsilon_{22}^{\left(1\right)}\chi_{2i}^{\left(1\right)}\right)B_{i}^{*}\gamma_{i}^{\left(1\right)},$$

$$e^{s\gamma y}=\sum_{i=1}^{2}e^{s\gamma_{i}^{\left(1\right)}y}$$

On the crack surface, there is

(19) $$\int_{a_{1}}^{b_{1}}(\left[\begin{array}{cc} C_{1}^{*} & C_{2}^{*}\\ C_{3}^{*} & C_{4}^{*} \end{array}\right]\frac{1}{B^{*}(f-x)}+\left[\begin{array}{cc} k_{1}(x,f) & k_{2}(x,f)\\ k_{3}(x,f) & k_{4}(x,f) \end{array}\right])\left[\begin{array}{c} h_{w}(f)\\ h_{\phi }(f) \end{array}\right]df=-\frac{\pi }{p}\left[\begin{array}{c} \sigma_{0}\\ D_{0} \end{array}\right]$$

where

$$k_{1}(x,f)=\int_{0}^{\infty }\left(\frac{C_{1}}{A^{*}}-\frac{C_{1}^{*}}{B^{*}}\right)\sin(s(f-x))ds,$$

$$k_{2}(x,f)=\int_{0}^{\infty }\left(\frac{C_{2}}{A^{*}}-\frac{C_{2}^{*}}{B^{*}}\right)\sin(s(f-x))ds,$$

$$k_{3}(x,f)=\int_{0}^{\infty }\left(\frac{C_{3}}{A^{*}}-\frac{C_{3}^{*}}{B^{*}}\right)\sin(s(f-x))ds,$$

$$k_{4}(x,f)=\int_{0}^{\infty }\left(\frac{C_{4}}{A^{*}}-\frac{C_{4}^{*}}{B^{*}}\right)\sin(s(f-x))ds.$$

The value of $C_{1}^{*},C_{2}^{*},C_{3}^{*},C_{4}^{*},B^{*}$ is referred to in Appendix A.

To simplify the calculation, we will transform the normalized quantity into the standard form and introduce the dislocation density function

$$h_{w}(x)=q_{1}(x),\;h_{\phi }(x)=q_{2}(x),\;k_{l}(x,f)=P_{l}(r,c),\;(l=1,2,3,4),$$

(20) $$m_{o}=\frac{b_{1}-a_{1}}{2},\;n_{o}=\frac{b_{1}+a_{1}}{2},\;f=\frac{x-n_{0}}{m_{0}},\;c=\frac{f-n_{0}}{m_{0}},$$

$$q_{1}(c)=\frac{h_{1}(c)}{\sqrt{1-c^{2}}}\pi \frac{\sigma_{0}}{p},\;q_{2}(c)=\frac{h_{2}(c)}{\sqrt{1-c^{2}}}\pi \frac{D_{0}}{p}.$$

According to Equation (20) and the Chebyshev placement method, Equation (19) can be transformed into

(21) $$\frac{1}{N}\sum_{Q=0}^{N}\Phi_{Q}(\left[\begin{array}{cc} C_{1}^{*} & C_{2}^{*}\\ C_{3}^{*} & C_{4}^{*} \end{array}\right]\Xi +m_{0}\left[\begin{array}{cc} P_{1}(r_{u},c_{Q}) & P_{2}(r_{u},c_{Q})\\ P_{3}(r_{u},c_{Q}) & P_{4}(r_{u},c_{Q}) \end{array}\right])\left[\begin{array}{c} h_{1}(c_{Q})\\ h_{2}(c_{Q}) \end{array}\right]=\left[\begin{array}{c} -1\\ -1 \end{array}\right]$$

where $\Xi =\frac{m_{0}}{B^{*}(m_{0}c_{Q}+n_{0}-m_{0}r_{u}-n_{0})}$, $\sum_{Q=0}^{N}\Phi_{Q}h_{1}(c_{Q})=0$, $\sum_{Q=0}^{N}\Phi_{Q}h_{2}(c_{Q})=0$, u=1,2,…N, u=1,2,…N, $\Phi_{0}=\Phi_{N}=\frac{1}{2}$, $\Phi_{1}=\ldots =\Phi_{N-1}=1$, $r_{u}=\cos\frac{(2u-1)\pi }{2N}$, $c_{Q}=\cos\frac{Q\pi }{N}$ (N represents a node).

## 4. Intensity Factor

According to Equation (21), it can be obtained that

(22) $$\underset{x\to a_{1}^{-}}{\lim}\sigma_{yz}^{*}(x,0,p)=\frac{\sigma_{0}C_{1}^{*}}{pB^{*}}\underset{c\to -1^{-}}{\lim}\frac{h_{1}(-1)}{\sqrt{c^{2}-1}}+\frac{\sigma_{0}C_{2}^{*}}{pB^{*}}\underset{c\to -1^{-}}{\lim}\frac{h_{2}(-1)}{\sqrt{c^{2}-1}}$$

(23) $$\underset{x\to b_{1}^{+}}{\lim}\sigma_{yz}^{*}(x,0,p)=\frac{\sigma_{0}C_{1}^{*}}{pB^{*}}\underset{c\to 1^{+}}{\lim}\frac{-h_{1}(1)}{\sqrt{c^{2}-1}}+\frac{\sigma_{0}C_{2}^{*}}{pB^{*}}\underset{c\to 1^{+}}{\lim}\frac{-h_{2}(1)}{\sqrt{c^{2}-1}}$$

(24) $$\underset{x\to a_{1}^{-}}{\lim}D_{y}^{*}(x,0,p)=\frac{D_{0}C_{3}^{*}}{pB^{*}}\underset{c\to -1^{-}}{\lim}\frac{h_{1}(-1)}{\sqrt{c^{2}-1}}+\frac{D_{0}C_{4}^{*}}{pB^{*}}\underset{c\to -1^{-}}{\lim}\frac{h_{2}(-1)}{\sqrt{c^{2}-1}}$$

(25) $$\underset{x\to b_{1}^{+}}{\lim}D_{y}^{*}(x,0,p)=\frac{D_{0}C_{3}^{*}}{pB^{*}}\underset{c\to 1^{+}}{\lim}\frac{-h_{1}(1)}{\sqrt{c^{2}-1}}+\frac{D_{0}C_{4}^{*}}{pB^{*}}\underset{c\to 1^{+}}{\lim}\frac{-h_{2}(1)}{\sqrt{c^{2}-1}}$$

Using Miller and Guy, assuming that the Laplace transform of f(t) is $f^{*}(p)$, p can be given by the following discrete points

(26) p=δ(β+1+k)

where δ>0, β>−1, k=0,1,2,⋯, Then it can be concluded that

(27) $$f(t)=\sum_{T=0}^{N}B_{T}P_{T}^{\left(0,\beta \right)}\left[2e^{-\delta t}-1\right],\;\delta >0,\;\beta >-1$$

Here, $P_{T}^{\left(0,\beta \right)}$ is a Jacobi polynomial, and the coefficients $B_{T}$ are determined by the following system of equations

(28) $$\delta f^{*}(\delta (\beta +1+k))=\sum_{m=0}^{k}\frac{k(k-1)\cdots \left[k-(m-1)\right]}{\left(k+\beta +1)(k+\beta +2)\cdots (k+\beta +1+m\right)}B_{T}$$

Based on the definitions of stress and electric displacement, the stress intensity factors $K_{III}$ and the electric displacement intensity factors $K_{IV}$ at the crack tips $x=a_{1}$ and $x=b_{1}$ can be obtained as

(29) $$\left\{\begin{array}{c} K_{III}(a_{1})\\ K_{IV}(a_{1}) \end{array}\right\}={\left(\sqrt{2\pi (a_{1}-x)}\right)}_{x\to a_{1}-0}\left\{\begin{array}{c} \sigma_{yz}(x,0,t)\\ D_{y}(x,0,t) \end{array}\right\}$$

(30) $$\left\{\begin{array}{c} K_{III}(b_{1})\\ K_{IV}(b_{1}) \end{array}\right\}={\left(\sqrt{2\pi (x-b_{1})}\right)}_{x\to b_{1}+0}\left\{\begin{array}{c} \sigma_{yz}(x,0,t)\\ D_{y}(x,0,t) \end{array}\right\}$$

Here, a non-dimensional function is defined

(31) $$K=K_{III}(t,v)/K_{III}(0,v)$$

Here, $K_{III}(0,v)$ represents the stress intensity factor under static load conditions.

## 5. Far-Field Forces and Electric Impact Loading

For the crack problem in piezoelectric media, as a cutting-edge branch of multi-physics field-coupled fracture mechanics, its core difficulty lies in accurately describing the complex boundary conditions at the crack tip and its surface. These conditions involve not only mechanical stress and strain but are also closely related to the electric field and electric displacement, forming a highly nonlinear boundary-value problem.

The classical strict electro-mechanical boundary conditions require that the normal displacement components at the surface of the piezoelectric medium (as the defect interface) and within the material must be continuous in the vicinity of the crack tip and at the crack interface, and the potential or electric field may also need to satisfy specific continuity or jump conditions. This strict condition stems from the principle of physical continuity and is currently the most accurate theoretical description. However, as stated in the original text, this greatly limits the scope of obtaining analytical solutions or closed-form solutions, making it extremely difficult to deeply understand and predict the fracture behavior of piezoelectric materials under complex conditions, severely restricting the application of related theories in engineering practice.

To overcome this challenge, the engineering and academic communities have developed a practical and effective approximation method. The core idea is to simplify the electric field based on the “transparency” of the crack surface. Currently, the most widely accepted and applied approach is to classify crack-boundary conditions into two categories: impermeable cracks and permeable cracks. The impermeable crack model, also known as the D-P boundary condition, is the most commonly used simplified model. It is based on a key assumption: the crack surface is completely insulated and does not allow any free charges, so the free charge density on the crack surface is zero. According to Gauss’s law, this means that the normal electric displacement component on the crack surface must be zero. Additionally, this model typically assumes that the potential on the crack surface remains constant. This boundary condition significantly simplifies the mathematical processing as it directly eliminates the influence of the crack surface as an electric field boundary, making the crack surface a “free surface” or an “equipotential surface”.

Although from a physical perspective this might be overly idealized (completely preventing the electric field from penetrating), in many engineering applications, especially when the crack propagation direction is not consistent with the electric field direction or when the crack size is much smaller than the characteristic wavelength, the D-P condition can provide results with reasonable accuracy, becoming a common benchmark for fracture mechanics analysis (such as calculating stress-intensity factors, electric-displacement-intensity factors) and structural-safety assessment. The permeable-crack model takes the opposite extreme, allowing the electric field to pass through the crack surface. In the permeable model, it is assumed that the potentials on the upper and lower surfaces of the crack are equal (i.e., the potential is continuous), or more generally, it allows for a certain jump in the potential on both sides of the crack surface, but the key is that the normal electric displacement components on both sides of the crack surface remain continuous. This means that the crack surface is no longer completely insulated but allows current to pass through. The permeable model typically better reflects the actual distribution of the electric field at the crack, especially in strong electric-field effects or specific geometric structures. However, its mathematical processing is usually more complex, and analytical solutions are more difficult to obtain.

By introducing these approximate boundary conditions, although the accuracy of the model is sacrificed to some extent, it significantly reduces the difficulty of solving the piezoelectric crack problem, enabling the use of analytical methods, semi-analytical methods to analyze fracture behavior, calculate stress intensity factors to be possible, and assess the safety of structures under complex load coupling. These approximate models provide important theoretical tools and engineering methods for understanding and predicting the failure mechanism of piezoelectric intelligent structures (such as piezoelectric sensors, detectors, and actuators).

In this study, we investigate the crack-growth problem subjected to stress and electric displacement impact loading at infinity, denoted by τ∞H(t) and D∞H(t), respectively. The boundary conditions described in Equations (5) and (7) are revised and transformed into the following forms

(32) $$\sigma_{yz}(x,d_{1},t)=\sigma_{yz}(x,-d_{2},t)=0$$

(33) $$w(x,0^{+},t)=w(x,0^{-},t),\;D_{y}(x,0^{+},t)=D_{y}(x,0^{-},t)$$

(34) $$\begin{array}{l} \sigma_{yz}(x,0^{+},t)=\sigma_{yz}(x,0^{-},t)=-\tau_{\infty }H(t)\\ D_{y}(x,0^{+},t)=D_{y}(x,0^{-},t)=D^{*}-D_{\infty }H(t) \end{array}$$

where $D^{*}$ is the electrical displacement in the crack plane. Take the Laplace transform of Equation (34), we obtain

(35) $$\sigma_{yz}^{*}(x,0^{+},p)=\sigma_{yz}^{*}(x,0^{-},p)=-\frac{\tau_{\infty }}{p},\;D_{y}^{*}(x,0^{+},p)=D_{y}^{*}(x,0^{-},p)=-\frac{D_{\infty }-D^{*}}{p}$$

### 5.1. Impermeable Crack (D-P)

For impermeable cracks, the electric potential in the crack cavity can be reasonably neglected. This is because the dielectric constant of piezoelectric materials is usually three to four orders of magnitude higher than that of air or vacuum. Therefore, the physical quantities appearing in Equation (35) $D^{*}$ meet the following conditions [23]:

(36) $$D^{*}=0$$

According to the aforementioned derivation process, the stress-intensity factor represented by $a_{1}$ and $b_{1}$ can be expressed as

(37) $$\underset{x\to a_{1}^{-}}{\lim}\sigma_{yz}^{*}(x,0,p)=\frac{\sigma_{\infty }C_{1}^{*}}{pB^{*}}\underset{c\to -1^{-}}{\lim}\frac{h_{1}(-1)}{\sqrt{c^{2}-1}}+\frac{\sigma_{\infty }C_{2}^{*}}{pB^{*}}\underset{c\to -1^{-}}{\lim}\frac{h_{2}(-1)}{\sqrt{c^{2}-1}}$$

(38) $$\underset{x\to b_{1}^{+}}{\lim}\sigma_{yz}^{*}(x,0,p)=\frac{\sigma_{\infty }C_{1}^{*}}{pB^{*}}\underset{c\to 1^{+}}{\lim}\frac{-h_{1}(1)}{\sqrt{c^{2}-1}}+\frac{\sigma_{\infty }C_{2}^{*}}{pB^{*}}\underset{c\to 1^{+}}{\lim}\frac{-h_{2}(1)}{\sqrt{c^{2}-1}}$$

(39) $$\underset{x\to a_{1}^{-}}{\lim}D_{y}^{*}(x,0,p)=\frac{D_{\infty }C_{3}^{*}}{pB^{*}}\underset{c\to -1^{-}}{\lim}\frac{h_{1}(-1)}{\sqrt{c^{2}-1}}+\frac{D_{\infty }C_{4}^{*}}{pB^{*}}\underset{c\to -1^{-}}{\lim}\frac{h_{2}(-1)}{\sqrt{c^{2}-1}}$$

(40) $$\underset{x\to b_{1}^{+}}{\lim}D_{y}^{*}(x,0,p)=\frac{D_{\infty }C_{3}^{*}}{pB^{*}}\underset{c\to 1^{+}}{\lim}\frac{-h_{1}(1)}{\sqrt{c^{2}-1}}+\frac{D_{\infty }C_{4}^{*}}{pB^{*}}\underset{c\to 1^{+}}{\lim}\frac{-h_{2}(1)}{\sqrt{c^{2}-1}}$$

In terms of the definitions of stress and electric displacement, the corresponding factors at the crack fronts can be formally derived as:

(41) $$\left\{\begin{array}{c} K_{III}(a_{1})\\ K_{IV}(a_{1}) \end{array}\right\}={\left(\sqrt{2\pi (a_{1}-x)}\right)}_{x\to a_{1}-0}\left\{\begin{array}{c} \sigma_{yz}(x,0,t)\\ D_{y}(x,0,t) \end{array}\right\}$$

(42) $$\left\{\begin{array}{c} K_{III}(b_{1})\\ K_{IV}(b_{1}) \end{array}\right\}={\left(\sqrt{2\pi (x-b_{1})}\right)}_{x\to b_{1}+0}\left\{\begin{array}{c} \sigma_{yz}(x,0,t)\\ D_{y}(x,0,t) \end{array}\right\}$$

According to Equations (41) and (42) and combined with the virtual-crack-closure technique, the corresponding energy release rate G can be obtained [24].

### 5.2. Permeable Crack

For the penetrating cracks, when the material in the text degenerates into an isotropic material, the coupling degree of the mechanical and electrical loads can be expressed as

(43) $$c_{55}^{\left(k\right)}(\frac{\partial^{2}w}{\partial X^{2}}+\frac{\partial^{2}w}{\partial Y^{2}})+e_{15}^{\left(k\right)}(\frac{\partial^{2}\phi }{\partial X^{2}}+\frac{\partial^{2}\phi }{\partial Y^{2}})=\rho \frac{\partial^{2}w}{\partial t^{2}}$$

(44) $$e_{15}^{\left(k\right)}(\frac{\partial^{2}w}{\partial X^{2}}+\frac{\partial^{2}w}{\partial Y^{2}})-\varepsilon_{11}^{\left(k\right)}(\frac{\partial^{2}\phi }{\partial X^{2}}+\frac{\partial^{2}\phi }{\partial Y^{2}})=0$$

(45) $$\frac{e_{15}D_{\infty }}{\varepsilon_{11}\tau_{\infty }}=\lambda$$

Note: In the previous calculation, $c_{55}^{\left(k\right)}=c_{44}^{\left(k\right)}$, $e_{15}^{\left(k\right)}=e_{24}^{\left(k\right)}$ and $\varepsilon_{11}^{\left(k\right)}=\varepsilon_{22}^{\left(k\right)}$.

For the permeable crack model, the continuity of the electric potential at the upper and lower surfaces of the crack is the core electrical boundary condition, which means that the electric potential of the media on both sides of the crack is equal everywhere on the crack surface

(46) $$h_{\phi }(x)=0$$

According to the aforementioned derivation process, the stress-intensity factor represented by $a_{1}$ and $b_{1}$ can be expressed as

(47) $$\underset{x\to a_{1}^{-}}{\lim}\sigma_{yz}^{*}(x,0,p)=\frac{\sigma_{\infty }C_{1}^{*}}{pB^{*}}\underset{c\to -1^{-}}{\lim}\frac{h_{1}(-1)}{\sqrt{c^{2}-1}}+\frac{\sigma_{\infty }C_{2}^{*}}{pB^{*}}\underset{c\to -1^{-}}{\lim}\frac{h_{2}(-1)}{\sqrt{c^{2}-1}}$$

(48) $$\underset{x\to b_{1}^{+}}{\lim}\sigma_{yz}^{*}(x,0,p)=\frac{\sigma_{\infty }C_{1}^{*}}{pB^{*}}\underset{c\to 1^{+}}{\lim}\frac{-h_{1}(1)}{\sqrt{c^{2}-1}}+\frac{\sigma_{\infty }C_{2}^{*}}{pB^{*}}\underset{c\to 1^{+}}{\lim}\frac{-h_{2}(1)}{\sqrt{c^{2}-1}}$$

(49) $$\underset{x\to a_{1}^{-}}{\lim}D_{y}^{*}(x,0,p)=\frac{\lambda \varepsilon_{11}\tau_{\infty }C_{3}^{*}}{e_{15}pB^{*}}\underset{c\to -1^{-}}{\lim}\frac{h_{1}(-1)}{\sqrt{c^{2}-1}}+\frac{\lambda \varepsilon_{11}\tau_{\infty }C_{4}^{*}}{e_{15}pB^{*}}\underset{c\to -1^{-}}{\lim}\frac{h_{2}(-1)}{\sqrt{c^{2}-1}}$$

(50) $$\underset{x\to b_{1}^{+}}{\lim}D_{y}^{*}(x,0,p)=\frac{\lambda \varepsilon_{11}\tau_{\infty }C_{3}^{*}}{e_{15}pB^{*}}\underset{c\to 1^{+}}{\lim}\frac{-h_{1}(1)}{\sqrt{c^{2}-1}}+\frac{\lambda \varepsilon_{11}\tau_{\infty }C_{4}^{*}}{e_{15}pB^{*}}\underset{c\to 1^{+}}{\lim}\frac{-h_{2}(1)}{\sqrt{c^{2}-1}}$$

## 6. Numerical Examples

To verify the above method and analyze its application, we simulated some numerical examples. In the following examples, a represents the half-length of the crack.

### 6.1. The Cracks Change in Accordance with the Influence of Elastic Constants

**Figure 2:**
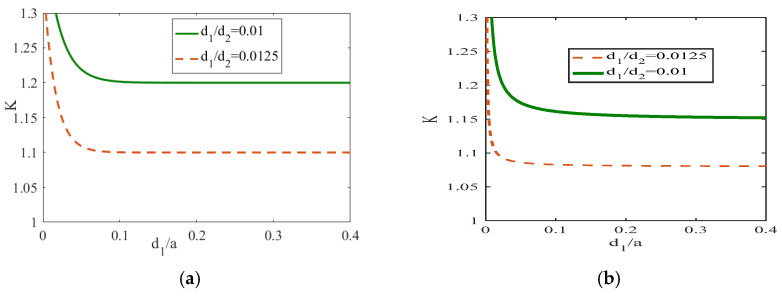
The variation of K with respect to $d_{1}/a$ ((**a**): $\varsigma_{c}=2$; (**b**): $\varsigma_{c}=1.5$).

The variation in the dimensionless function with different elastic constant ratios is shown in Figure 2a,b. When the elastic constant ratio $\varsigma_{c}=c_{44}^{\left(2\right)}/c_{44}^{\left(1\right)}$ was different, the piezoelectric constant ratio and dielectric constant ratio were both 1. The crack changes under different thickness ratios of materials 1 and 2 ($d_{1}/d_{2}=0.0125$ and $d_{1}/d_{2}=0.01$) were analyzed respectively in the cases of elastic constant ratio $\varsigma_{c}=2$ (Figure 2a) and $\varsigma_{c}=1.5$ (Figure 2b).

As can be seen from the figure, the non-dimensional function decreases continuously as $d_{1}/a$ increases and gradually approaches a stable value. Moreover, when the elastic modulus is large, the stable value of the non-dimensional function is also larger. At the same time, it can be observed that when the thickness of material 2 is fixed, the larger the thickness of material 1, the smaller the influence on the non-dimensional function. That is, the smaller the thickness of material 1, the greater the stress intensity generated. Therefore, in application, if we make material 1 relatively thinner, it will have higher practical application value.

### 6.2. The Variation in Crack Under Impact Time and Crack Length

**Figure 3:**
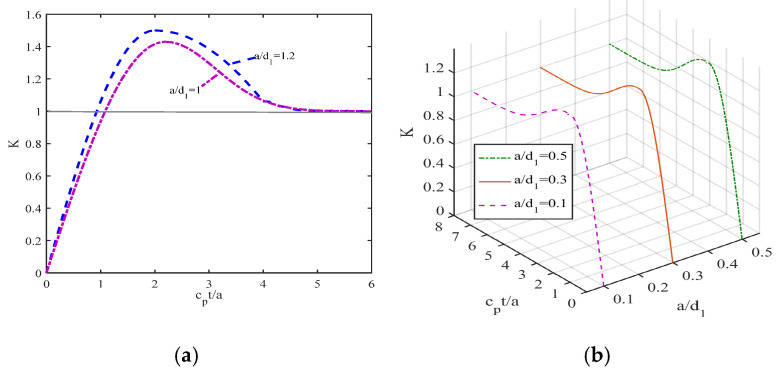
(**a**) The 2D plot that K varies with $c_{p}t/a$; (**b**) The three-dimensional graph that K varies with $c_{p}t/a$.

It can be clearly seen from the two-dimensional and three-dimensional curves presented in Figure 3a,b that the dimensionless dynamic stress intensity factor at the crack tip of the piezoelectric bimaterial interface shows obvious transient dynamic behaviors under impact load. Over time, the dimensionless function first rises rapidly, reaching a significant peak within a short period of time, then begins to gradually decline, presenting small oscillations within a certain range, and finally converges gradually to the corresponding stress intensity factor value under the static load.

This change process fully demonstrates the comprehensive influence of stress-wave propagation, reflection, superposition, and energy dissipation under impact load on the mechanical field at the crack tip and also reflects the significant differences between dynamic fracture behavior and quasi-static fracture behavior. The appearance of the peak indicates that in the initial stage of impact, the stress wave focuses strongly at the crack tip, resulting in a significant enhancement of the local mechanical and electric field coupling effect, while the oscillation convergence in the later stage indicates that the system gradually stabilizes and the dynamic effect gradually decays.

At the same time, the numerical results also show that, while keeping the thickness of material 1 unchanged, the crack length has a significant positive regulatory effect on the dimensionless function. As the crack length increases, the dimensionless dynamic stress intensity factor shows a significant increasing trend overall, which means that the expansion of the crack size will further intensify the stress concentration at the interface and increase the risk of dynamic expansion and unstable fracture of the crack.

These laws not only reveal the intrinsic relationship between geometric parameters, load forms and dynamic fracture behavior, but also provide important theoretical basis and data support for the strength design, damage identification and safety assessment of piezoelectric composite material structures under complex dynamic conditions such as impact and vibration.

### 6.3. Impermeable and Permeable Cracks

**Figure 4:**
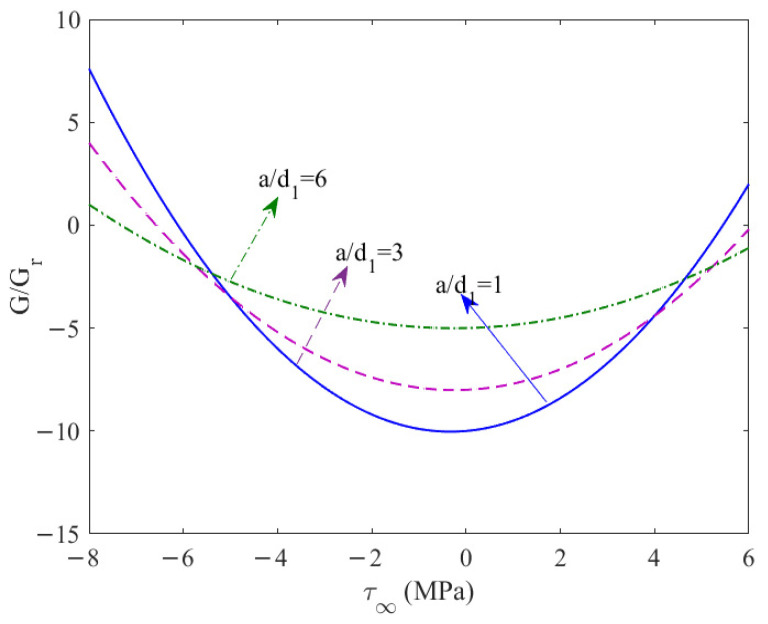
The variation in the normalized energy release rate under far-field stress loading.

The variation in the normalized energy-release rate under far-field stress loading is shown in Figure 4. The dynamic nondimensional energy-release rate can be expressed as $G/G_{r}$ ($G_{r}$ is the dynamic energy-release rate for a stationary crack). Figure 4 shows that when the far-field shear stress $\tau_{\infty }$ increases from zero, the normalized energy release rate $G/G_{r}$ always shows an upward trend. However, when $\tau_{\infty }$ decreases from zero (i.e., in a negative shear stress state), $G/G_{r}$ first decreases and then increases. This phenomenon demonstrates that the effects of positive and negative shear stresses on crack propagation have significant asymmetry: positive shear stress can continuously increase the energy release rate, thereby promoting crack propagation, while negative shear stress will reduce the energy release rate within a certain small range, manifesting as inhibition or hindrance of crack development. However, as the absolute value of negative shear stress further increases, the energy release rate will rise again. Thus, stress loading can either promote crack propagation or have interference or blocking effects, the specific manifestation depending on the direction and magnitude of the far-field stress. The above change pattern is completely consistent with the theoretical analysis results established by Pak [23], further verifying the rationality of the calculation model and the shear fracture theory presented in this paper.

**Figure 5:**
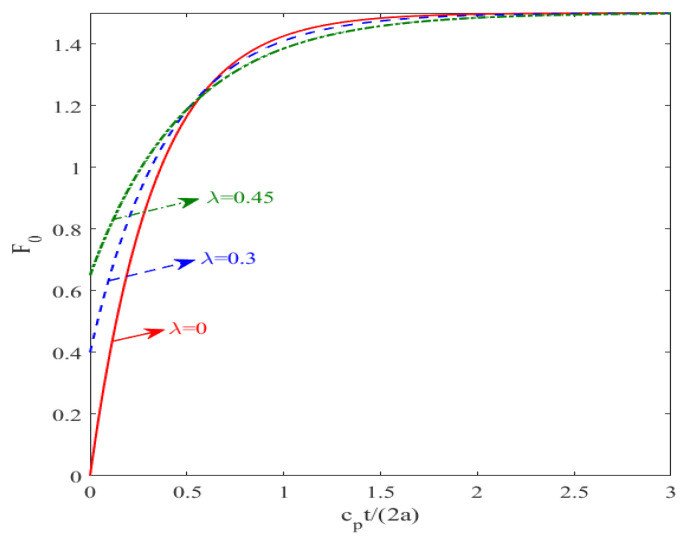
The variation in the normalized intensity factor under different λ values.

In order to conduct a more in-depth study on the dynamic crack propagation under the coupling impact of mechanics and electricity, Figure 5 presents the evolution of the normalized dynamic strength factor under different electromagnetic–mechanical coupling coefficients λ. It is clearly observable that as the parameter ct/(2a) related to time increases, the normalized dynamic strength factor $F_{0}$ first rises rapidly, reaching a significant peak, then gradually decreases and eventually stabilizes at a constant value. This characteristic trend, including the sharp rise, peak response, and final steady-state behavior, is highly consistent with the dynamic fracture response studied by Wang [25].

### 6.4. Response-Surface Statistical Analysis

This study employed Response Surface Methodology (RSM), a statistical technique that integrates experimental design, modeling, and optimization techniques, aiming to systematically explore and quantify the influence of multiple controllable factors on a specific response variable, and ultimately determine the conditions for obtaining the optimal response. In this study, we specifically focused on the influence pattern of three key factors—Factor A ($d_{1}/a$), Factor B ($c_{p}t/a$), and Factor C ($c_{44}^{\left(2\right)}/c_{44}^{\left(1\right)}$)—on the Stress Intensity Factor R1 (SIF) response variable.

First, to efficiently and comprehensively obtain data points for modeling, we adopted the Box–Behnken Design (BBD) experimental design method provided by Design-Expert software V22. BBD is a commonly used response surface design method, particularly suitable for situations where it is necessary to estimate the interaction and quadratic effects between factors.

Next, we used the statistical module of Design-Expert to perform a quadratic polynomial regression fit on the data. To assess the reliability and explanatory power of the established model, we conducted rigorous statistical analyses. The model-fitting goodness was measured by the coefficient of determination R^2^ (R-squared) and the adjusted coefficient of determination Adjusted R^2^. The R^2^ value represents the proportion of the total variation in the response variable explained by the model; the closer it is to 1, the better the model fit. Adjusted R^2^ considers the number of independent variables in the model and adjusts R^2^, providing a more realistic reflection of the model’s actual predictive ability. At the same time, we performed an Analysis of Variance (ANOVA). Analysis of Variance (ANOVA) decomposes the contribution of independent variables to the total variation to assess the impact of each factor on the response variable (Stress Intensity Factor R1). The significance of each factor on the response value is mainly achieved by comparing the variance between groups with that within groups, thereby evaluating the accuracy and reliability of the model. The significance of the model is tested by analyzing the sources of error, and the F-value and *p*-value are combined for joint judgment. The larger the F value and the smaller the *p* value, the more significant the model is. When the *p* value is greater than 0.05, the model is not significant. When the *p*-value is less than 0.05, the model is significant, especially when the *p*-value is less than 0.0001, the model shows extremely high significance. According to the data in Table 1, the *p* value is less than 0.0001, so the model is significant. The ANOVA results not only validated the statistical significance of the entire model (through F-test, typically requiring a *p*-value < 0.05) but also tested the significance of individual coefficients (linear, quadratic, interaction terms) (through *t*-test, typically requiring a *p*-value < 0.05 or 0.01), helping us understand which factors and their interactions significantly affect SIF. Residual analysis was also conducted to further validate the model’s predictive ability and the reasonableness of its assumptions.

Finally, based on the established and validated quadratic response surface model, we utilized the optimization tools built into the Design-Expert software to perform optimization calculations on the combination of Factors A, B, and C. The optimization objective was set to maximize the Stress Intensity Factor (SIF) response value R1. The optimization process considered all significant factor effects (including linear, quadratic, and interaction effects) and may have set operational constraints on the factors (such as factors not exceeding their physical or process-allowable ranges). The optimization algorithm in Design-Expert searched the factor space to find the optimal combination of Factors A, B, and C that maximizes SIF under the constraint conditions. This optimal combination not only provides clear guidance for practical applications but also intuitively demonstrates the core value of RSM—namely, finding optimal operating conditions from complex factor interactions through mathematical modeling and optimization.

The results of the analysis of variance (as shown in Table 1 and Table 2) show that the F value of the regression model is 706.4, and the *p* values of each model are all less than 0.05, indicating the significance of the model. Among them, the *p* values of A-A, B-B, A^2^, B^2^, and C^2^ are all less than 0.0001, indicating that the model is extremely significant and highly reliable, and the experimental design is reasonable. Therefore, the results of the analysis of variance indicate that the fitting model is accurate and statistically significant.

According to the data, a second-order response surface mathematical model was obtained through analysis. The second-order response surface regression model regarding the three variables A, B, and C, as well as R1, is shown in Equation (51).

(51) $$\begin{array}{c} R1=-12.2865+0.8313A+1.5650B+14.0871C+0.1817\mathrm{AB}+0.1818\mathrm{AC}-0.0691\mathrm{BC}\\ -0.8666A^{2}-0.4544B^{2}-4.07760C^{2} \end{array}$$

**Figure 6:**
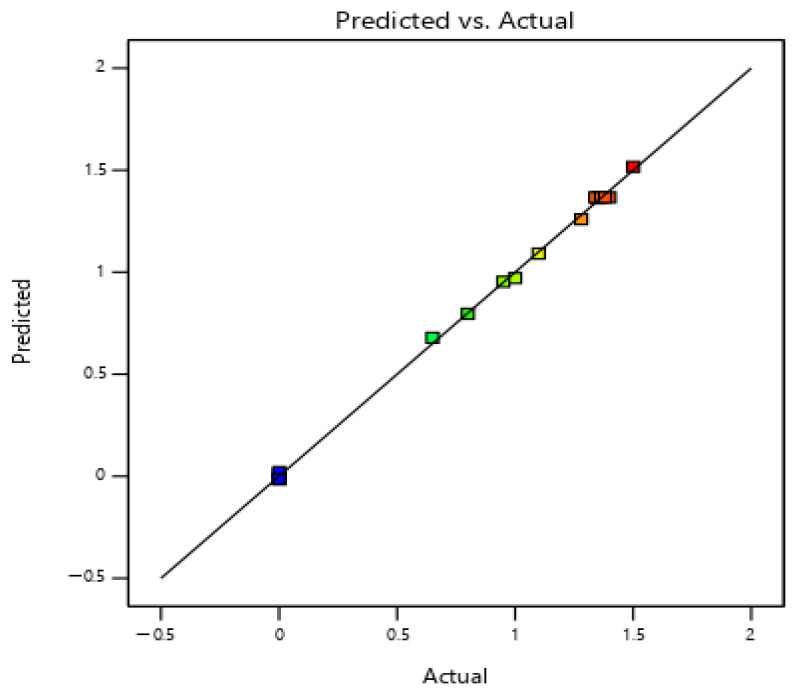
Comparison chart of real values and predicted values.

Figure 6 depicts the relationship between the predicted values and the actual values. The horizontal axis represents the actual values, and the vertical axis represents the predicted values. Under ideal conditions, if the predicted value is equal to the actual value, it lies on the black straight line. Different data points are represented by squares of different colors. By observing the image, it can be found that the vast majority of data points are concentrated near the black straight line, which directly indicates that the predicted values are close to the actual values. This also shows that the established prediction model has good accuracy and reliability, can predict the relevant values relatively accurately, and can effectively reflect the true relationship between the variables.

It can be seen from Table 2 that the interaction terms AC and BC are statistically insignificant. This does not mean that there are no interaction effects at the physical level, but reflects that the strengths of these interactions are weaker compared with the main effects of factors A, B, and C. During the experiments, the coupled electromechanical effects corresponding to AC and BC do exist objectively, but their influences on the response variables (e.g., interfacial electromechanical coupling strength, stress–electric coupling distribution at the crack tip, etc.) are overshadowed by the dominant main effects. For example, A (thickness of Material 1/crack length) directly determines the constraint effect of Material 1 on crack propagation, and C (ratio of elastic constants of Material 2 to Material 1) mainly affects the mechanical matching across the interface. Their independent effects on the electromechanical coupling are more significant, whereas their synergistic regulation only induces marginal changes. In addition, we have verified the residual distribution of the model and found no systematic deviation, indicating that the insignificance of AC and BC does not result from improper model specification, but truly reflects the weak intensity of these interactions.

From the perspective of physical mechanism, the coupled electromechanical effects at the interface are essentially a complex process involving multi-factor synergy. According to the variable definitions, the synergistic pathways of AC and BC are inherently constrained by the geometrical parameters and mechanical properties of the materials, limiting their interactive contributions to a small range. Specifically, for the interaction term BC, the variation in crack propagation length is directly related to the stress concentration at the crack tip, while the elastic constant ratio determines the stress-transfer efficiency across the interface. During crack propagation, the regulation of stress transfer by the elastic constant ratio is overshadowed by the stress-concentration effect at the crack tip, producing only weak local effects that cannot be manifested as statistically significant changes in the macroscopic response variables. For the interaction term AC, their synergistic effect theoretically affects the distribution of interfacial electromechanical coupling. However, when the thickness of Material 1 is sufficient to form a stable load-bearing structure, the contribution of the interaction to the response variable becomes weak. Due to its small amplitude, it does not reach the threshold of statistical significance in the macroscopic response model, thus showing statistical insignificance.

In summary, the statistical insignificance of the interaction terms AC and BC is a reasonable reflection of the coupled electromechanical effects at the interface under the constraints of specific geometrical parameters and mechanical properties, rather than a contradiction with physical reality. The key to reconciling them is to clarify that “statistical significance” depends on the detectability of effects within the experimental design and modeling framework, while “physical existence” refers to the inherent synergistic behavior of the factors. These two concepts are complementary rather than mutually exclusive.

**Figure 7:**
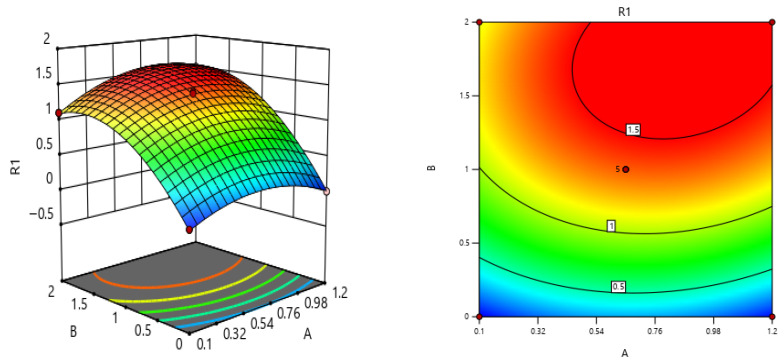
The three-dimensional graph and contour map of the interaction between A and B in terms of the influence on the stress intensity factor.

Through response-surface analysis of the parameters, A 3D response-surface map (as shown in Figure 7) and a contour map (as shown in Figure 7) were obtained. As shown in Figure 7, in the three-dimensional response-surface plot, the horizontal axis represents parameters A and B respectively, and the vertical axis represents the corresponding response values. The shape and gradient changes in the response surface intuitively reflect the variation pattern of the response values under the combined effect of the two factors: the red area corresponds to higher response values, the blue area corresponds to lower response values, and the degree of color gradient clearly shows the increase or decrease range of the response values.

In the contour plot, the same pattern is also presented, that is, the color that is closer to red indicates a higher response value, and the color that is closer to blue indicates a lower response value. Overall, the response-surface plot and the contour plot can intuitively, comprehensively and quantitatively reveal the spatial distribution characteristics, interaction influence rules and extreme distribution areas of the response values with respect to parameters A and B, and can provide reliable visual evidence and theoretical support for the subsequent identification of key parameters, analysis of the influencing mechanism and selection of the optimal parameter range.

**Figure 8:**
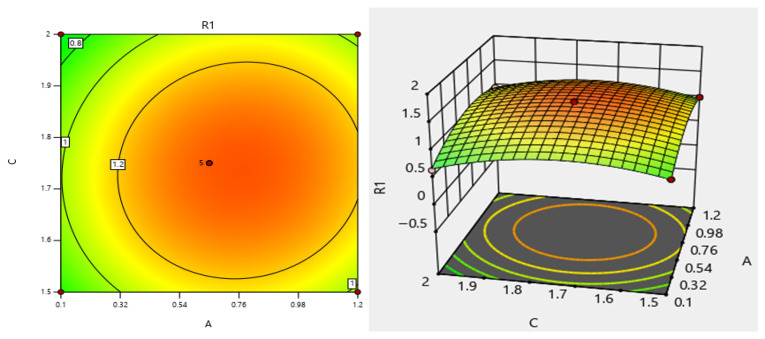
The three-dimensional graph and contour map of the interaction between A and C in terms of the influence on the stress-intensity factor.

In the three-dimensional response-surface plot, the horizontal axis is A and C, and the vertical axis R1 represents the obtained response values (as shown in Figure 8). This surface is used to reflect the continuous pattern of response values changing with the two independent variables. The steeper the surface, the stronger the influence of the corresponding independent variable on the response value in that area. If the surface is flat, the influence of the variable on the response value is relatively weak. For the contour plot, the color represents the gradual change (green-yellow-red), indicating high or low response values. The red area has the highest response value, while the green area has the lowest response value. Points on the same curve have the same response value. The denser the curve, the more intense the change in the response value in that area. Within the 1.5 range, the contours are elliptical, indicating a significant interaction between the two independent variables and the change in response values.

**Figure 9:**
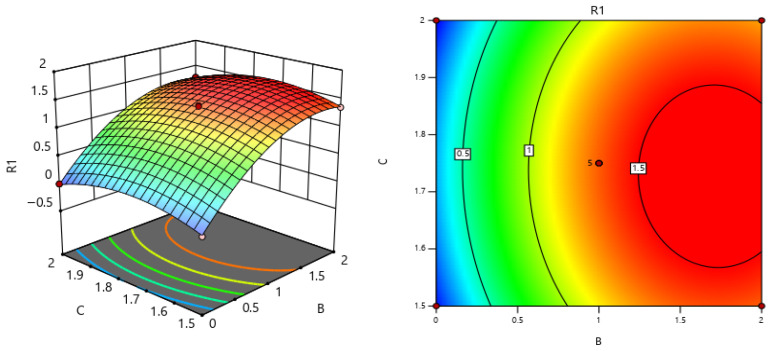
The three-dimensional graph and contour map of the interaction between B and C in terms of the influence on the stress intensity factor.

Figure 9 shows the three-dimensional response-surface plot and contour plot corresponding to the joint variation in independent variables B and C. From the figure, it can be seen that the response value shows a continuous and smooth trend as variables B and C change. The color in the figure gradually transitions from blue to red, intuitively reflecting the distribution pattern of the response value from low to high. The response surface as a whole presents a morphological characteristic of rapid increase first and then tending to be stable. This indicates that when B and C increase to a certain range, the growth rate of the response value gradually slows down and tends to stabilize.

Further analysis reveals that when the value of variable B is within the range of 1.5 to 2, the response value R1 can reach the optimal range of 1.8 to 2. At the same time, from the contour plot, a significant stretched elliptical distribution feature can be clearly observed. This feature directly indicates that there is a significant interaction between independent variables B and C, and their influence on the response value is not independent of each other but shows a clear coupling effect.

**Figure 10:**
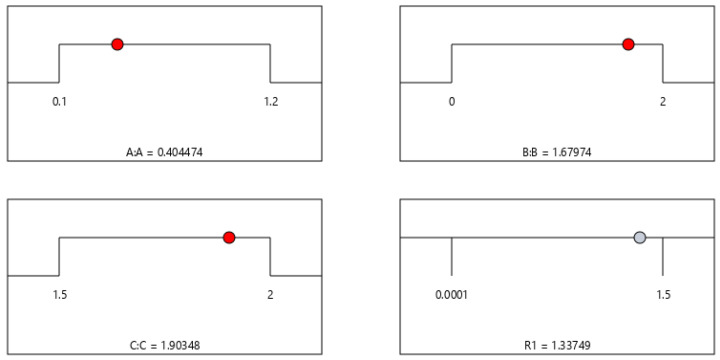
The response values of A, B and C when the stress-intensity factor reaches its maximum value (The red and grey dots represent the corresponding data values).

Depicted in Figure 10 are the response values of A, B, and C under the maximum stress intensity factor condition. From the figure, it can be seen that the optimal values of A, B and C are 0.4045, 1.6797 and 1.9035 respectively, and the stress intensity factor reaches its maximum value of 1.3375 at these values. Additionally, it is found that the interaction between B and C is the most significant. The combination of B and C has a more significant impact on the response value than the combinations of other factors.

Figure 10 shows the parameter combination when the stress-intensity factor reaches its global maximum: the corresponding values of variables A, B, and C are 0.4045, 1.6797, and 1.9035, respectively, at which the peak stress intensity factor is 1.3375. Meanwhile, the response surface analysis reveals that the interaction effect between factors B and C is statistically significant (*p* < 0.05), and their coupled influence on the stress intensity factor is significantly stronger than that of other factor combinations (see Table 2 for the significance analysis of interaction terms).

It should be clearly defined that the term “optimal parameters” here refers only to the mathematical extreme solution from the response-surface model, specifically the parameter point that maximizes the stress-intensity factor, rather than the optimal design scheme based on structural safety criteria. As a key parameter characterizing the stress-field intensity at the crack tip, the magnitude of the stress-intensity factor directly determines the crack initiation threshold and propagation rate. A higher stress-intensity factor indicates a more severe stress concentration at the crack tip, a greater probability of brittle fracture or fatigue failure, and lower structural safety. Therefore, the parameter combination shown in Figure 9 corresponds to the most unfavorable service condition of the structure, whose core value is to provide a boundary reference for dangerous working conditions in engineering design, rather than a recommended safe parameter range.

In practical structural safety design and fracture control, the high-risk parameter combination shown in Figure 9 should be avoided. We have supplemented the distinction between mathematically optimal extreme values and engineering safety-optimized solutions in the revised manuscript to prevent confusion of academic concepts, ensure the consistency between research conclusions and engineering practice, and thus provide a more valuable reference for structural safety design.

## 7. Conclusions

This paper conducts a systematic study on the dynamic propagation of orthogonal anisotropic interface cracks in piezoelectric bimaterial structures. By introducing Laplace transform and Fourier transform to convert the governing equations and combining the Chebyshev collocation method to discretize the original problem into algebraic equations, the dynamic variation laws of the stress intensity factor at the crack tip and the electric displacement intensity factor are obtained after numerical inverse transformation. Based on this, the influence mechanism of key parameters on the fracture behavior is analyzed by using the response surface method, and the following main conclusions are obtained:

(1) The elastic parameters have a significant regulatory effect on the dynamic propagation of interface cracks. Within a certain range, the larger the elastic parameters, the higher the dimensionless dynamic stress-intensity factor at the crack tip, indicating that the elastic properties of the material directly affect the degree of stress concentration and the driving ability of the crack.
(2) The impact-load application time significantly changes the dynamic response characteristics of the crack. With the passage of time, the dimensionless stress-intensity factor shows a pattern of first increasing, reaching a peak, and then oscillating and converging, eventually approaching the corresponding value under static load, reflecting the combined effect of stress wave propagation, reflection, and energy dissipation.
(3) The crack length is an important geometric factor affecting the fracture risk. The larger the crack length, the more significant the stress concentration at the tip, and the corresponding dimensionless dynamic stress-intensity factor increases. The structural fracture failure risk is higher.
(4) For non-permeable cracks, the dual effect of forward shear stress promoting crack propagation and negative shear stress first inhibiting and then driving the crack propagation has verified the rationality of the shear-fracture calculation model established in this paper. For the dimensionless dynamic strength factors corresponding to different force–electric coupling coefficients λ, they all show a typical characteristic of rapid increase—reaching a peak—slow decrease—approaching stability as $c_{p}t/\left(2a\right)$ changes. This further proves that the model in this paper can accurately describe the transient fracture-mechanics behavior of piezoelectric materials under coupled loads.
(5) The constructed prediction model has high accuracy and strong reliability, with a determination coefficient R^2^ of 0.9886. The real values and predicted values are highly consistent, providing an efficient and accurate data-driven method for the rapid prediction of the stress-intensity factor at the crack tip.
(6) The key parameter combinations that maximize the stress-intensity factor were obtained through response surface analysis: A = 0.4045, B = 1.6797, C = 1.9035, corresponding to a peak stress intensity factor of 1.3375. Significance analysis indicates that there is a significant interaction between factors B and C (*p* < 0.05), and their coupling effect is stronger than other parameter combinations.
(7) The “optimal parameter combination” obtained in this paper is only a mathematical extremum point. Even the parameter combination that maximizes the stress-intensity factor corresponds to the most dangerous service condition of the structure, rather than an engineering safety optimization scheme. This result can be used to identify high-risk parameter intervals, providing important theoretical references for the fracture prevention, safety design, and boundary determination of dangerous conditions of engineering structures.

Overall, the analytical–numerical combined analysis framework established in this paper not only enriches the dynamic fracture theory of piezoelectric bimaterial interface cracks but also provides feasible methods for the safety assessment, parameter optimization, and failure prevention of intelligent materials and structures. It has certain theoretical value and practical significance for promoting the reliable application of piezoelectric structures in engineering fields. In future research, we will incorporate the thickness ratio (d_1_/d_2_) as a key parameter into our analysis and conduct systematic quantitative studies using appropriate numerical simulation software. This extension aims to more comprehensively reveal the influence law of the thickness ratio on the dynamic fracture behavior of piezoelectric bimaterial interface cracks, further improving the completeness and engineering applicability of the proposed theoretical framework.

## Figures and Tables

**Table 1:** Variance analysis of predictive models.

Standard Error	Correlation Coefficient	Correction Coefficient	F	*p*	Significant
0.0285	0.9975	0.9886	706.4	<0.0001	significant

**Table 2:** Variance analysis of predictive model of response value.

Source	Sum of Square	Df	Mean Square	F-Value	*p*-Value	Significant
A-A	0.1013	1	0.1013	125.03	<0.0001	significant
B-B	3.42	1	3.42	4214.47	<0.0001	significant
C-C	0.0092	1	0.0092	11.32	0.0120	
AB	0.0399	1	0.0399	49.29	0.0002	
AC	0.0025	1	0.0025	3.09	0.1224	
BC	0.0012	1	0.0012	1.47	0.2642	
A^2^	0.2894	1	0.2894	357.08	<0.0001	significant
B^2^	0.8693	1	0.8693	1072.74	<0.0001	significant
C^2^	0.2735	1	0.2735	337.47	<0.0001	significant
Residual	0.0057	7	0.0008			
Lack of Fit	0.0034	3	0.0011	2.06	0.2480	not significant
Pure Error	0.0022	4	0.0006			
Cor Total	5.16	16				

## Data Availability

The original contributions presented in this study are included in the article, further inquiries can be directed to the corresponding author.

## Appendix A

$$\varpi_{1}^{\left(k\right)}=(-\frac{\varepsilon_{22}^{\left(k\right)}}{c_{44}^{\left(k\right)}}((\frac{c_{55}^{\left(k\right)}}{c_{44}^{\left(k\right)}}-\frac{v^{2}}{{\bar{c_{k}}}^{2}})s^{2}+\frac{2vpsi}{{\bar{c_{k}}}^{2}}+\frac{p^{2}}{{\bar{c_{k}}}^{2}})-\frac{2e_{24}^{\left(k\right)}e_{15}^{\left(k\right)}s^{2}}{c_{44}^{\left(k\right)}c_{44}^{\left(k\right)}}-\frac{\varepsilon_{11}^{\left(k\right)}s^{2}}{c_{44}^{\left(k\right)}})/((\frac{\varepsilon_{22}^{\left(k\right)}}{c_{44}^{\left(k\right)}}+{\left(\frac{e_{24}^{\left(k\right)}}{c_{44}^{\left(k\right)}}\right)}^{2}),$$

$$\varpi_{2}^{\left(k\right)}=(\frac{\varepsilon_{11}^{\left(k\right)}}{c_{44}^{\left(k\right)}}((\frac{c_{55}^{\left(k\right)}}{c_{44}^{\left(k\right)}}-\frac{v^{2}}{{\bar{c_{k}}}^{2}})s^{4}+\frac{2vps^{3}i}{{\bar{c_{k}}}^{2}}+\frac{p^{2}s^{2}}{{\bar{c_{k}}}^{2}})+\frac{e_{15}^{\left(k\right)}e_{15}^{\left(k\right)}s^{4}}{c_{44}^{\left(k\right)}c_{44}^{\left(k\right)}})/((\frac{\varepsilon_{22}^{\left(k\right)}}{c_{44}^{\left(k\right)}}+{\left(\frac{e_{24}^{\left(k\right)}}{c_{44}^{\left(k\right)}}\right)}^{2}),$$

$$\vartheta_{1}^{\left(k\right)}=\sqrt[{3}]{-\frac{72\varpi_{1}^{\left(k\right)}\varpi_{2}^{\left(k\right)}-2\varpi_{1}^{{\left(k\right)}^{3}}}{54}+\sqrt{\frac{{\left(72\varpi_{1}^{\left(k\right)}\varpi_{2}^{\left(k\right)}-2\varpi_{1}^{{\left(k\right)}^{3}}\right)}^{2}}{2916}-\frac{{\left(12\varpi_{2}^{\left(k\right)}+\varpi_{1}^{{\left(k\right)}^{2}}\right)}^{3}}{729}}},$$

$$\vartheta_{1}^{\left(k\right)}=\sqrt[{3}]{-\frac{72\varpi_{1}^{\left(k\right)}\varpi_{2}^{\left(k\right)}-2\varpi_{1}^{{\left(k\right)}^{3}}}{54}-\sqrt{{\left(\frac{72\varpi_{1}^{\left(k\right)}\varpi_{2}^{\left(k\right)}-2\varpi_{1}^{{\left(k\right)}^{3}}}{54}\right)}^{2}-{\left(\frac{12\varpi_{2}^{\left(k\right)}+\varpi_{1}^{{\left(k\right)}^{2}}}{9}\right)}^{3}}},$$

$$\gamma_{1}^{\left(k\right)}=\frac{-\sqrt{-2\varpi_{1}^{\left(k\right)}/3+\vartheta_{1}^{\left(k\right)}+\vartheta_{2}^{\left(k\right)}}+\sqrt{-4\varpi_{1}^{\left(k\right)}/3-\vartheta_{1}^{\left(k\right)}-\vartheta_{2}^{\left(k\right)}}}{2},$$

$$\gamma_{2}^{\left(k\right)}=\frac{-\sqrt{-2\varpi_{1}^{\left(k\right)}/3+\vartheta_{1}^{\left(k\right)}+\vartheta_{2}^{\left(k\right)}}-\sqrt{-4\varpi_{1}^{\left(k\right)}/3-\vartheta_{1}^{\left(k\right)}-\vartheta_{2}^{\left(k\right)}}}{2},$$

$$\gamma_{3}^{\left(k\right)}=\frac{\sqrt{-2\varpi_{1}^{\left(k\right)}/3+\vartheta_{1}^{\left(k\right)}+\vartheta_{2}^{\left(k\right)}}+\sqrt{-4\varpi_{1}^{\left(k\right)}/3-\vartheta_{1}^{\left(k\right)}-\vartheta_{2}^{\left(k\right)}}}{2},$$

$$\gamma_{4}^{\left(k\right)}=\frac{\sqrt{-2\varpi_{1}^{\left(k\right)}/3+\vartheta_{1}^{\left(k\right)}+\vartheta_{2}^{\left(k\right)}}-\sqrt{-4\varpi_{1}^{\left(k\right)}/3-\vartheta_{1}^{\left(k\right)}-\vartheta_{2}^{\left(k\right)}}}{2},$$

$$\begin{array}{c} A^{*}=(c_{44}^{\left(2\right)}\chi_{11}^{\left(2\right)}+e_{24}^{\left(2\right)}\chi_{21}^{\left(2\right)})\gamma_{1}^{\left(2\right)}e^{s\gamma_{1}^{\left(2\right)}d_{2}}(-\chi_{11}^{\left(1\right)}\chi_{22}^{\left(2\right)}+\chi_{12}^{\left(2\right)}\chi_{21}^{\left(1\right)}+\chi_{12}^{\left(1\right)}\chi_{22}^{\left(2\right)}-\chi_{12}^{\left(2\right)}\chi_{22}^{\left(1\right)})\\ +(c_{44}^{\left(2\right)}\chi_{12}^{\left(2\right)}+e_{24}^{\left(2\right)}\chi_{22}^{\left(2\right)})\gamma_{2}^{\left(2\right)}e^{s\gamma_{2}^{\left(2\right)}d_{2}}(-\chi_{11}^{\left(2\right)}\chi_{21}^{\left(1\right)}+\chi_{11}^{\left(1\right)}\chi_{21}^{\left(2\right)}-\chi_{12}^{\left(1\right)}\chi_{21}^{\left(2\right)}+\chi_{11}^{\left(2\right)}\chi_{22}^{\left(1\right)}) \end{array},$$

$$A_{1}=-(c_{44}^{\left(2\right)}\chi_{11}^{\left(2\right)}+e_{24}^{\left(2\right)}\chi_{21}^{\left(2\right)})\gamma_{1}^{\left(2\right)}e^{s\gamma_{1}^{\left(2\right)}d_{2}}\chi_{22}^{\left(2\right)}+(c_{44}^{\left(2\right)}\chi_{12}^{\left(2\right)}+e_{24}^{\left(2\right)}\chi_{22}^{\left(2\right)})\gamma_{2}^{\left(2\right)}e^{s\gamma_{2}^{\left(2\right)}d_{2}}\chi_{21}^{\left(2\right)},$$

$$B_{1}=-(c_{44}^{\left(2\right)}\chi_{12}^{\left(2\right)}+e_{24}^{\left(2\right)}\chi_{22}^{\left(2\right)})\gamma_{2}^{\left(2\right)}e^{s\gamma_{2}^{\left(2\right)}d_{2}}\chi_{11}^{\left(2\right)}+(c_{44}^{\left(2\right)}\chi_{11}^{\left(2\right)}+e_{24}^{\left(2\right)}\chi_{21}^{\left(2\right)})\gamma_{1}^{\left(2\right)}e^{s\gamma_{1}^{\left(2\right)}d_{2}}\chi_{12}^{\left(2\right)},$$

$$A_{2}=-(c_{44}^{\left(2\right)}\chi_{12}^{\left(2\right)}+e_{24}^{\left(2\right)}\chi_{22}^{\left(2\right)})\gamma_{2}^{\left(2\right)}e^{s\gamma_{2}^{\left(2\right)}d_{2}}\chi_{21}^{\left(2\right)}+(c_{44}^{\left(2\right)}\chi_{11}^{\left(2\right)}+e_{24}^{\left(2\right)}\chi_{21}^{\left(2\right)})\gamma_{1}^{\left(2\right)}e^{s\gamma_{1}^{\left(2\right)}d_{2}}\chi_{22}^{\left(2\right)},$$

$$B_{2}=-(c_{44}^{\left(2\right)}\chi_{11}^{\left(2\right)}+e_{24}^{\left(2\right)}\chi_{21}^{\left(2\right)})\gamma_{1}^{\left(2\right)}e^{s\gamma_{1}^{\left(2\right)}d_{2}}\chi_{12}^{\left(2\right)}+(c_{44}^{\left(2\right)}\chi_{12}^{\left(2\right)}+e_{24}^{\left(2\right)}\chi_{22}^{\left(2\right)})\gamma_{2}^{\left(2\right)}e^{s\gamma_{2}^{\left(2\right)}d_{2}}\chi_{11}^{\left(2\right)},$$

$$A_{1}^{*}=(c_{44}^{\left(2\right)}\chi_{12}^{\left(2\right)}+e_{24}^{\left(2\right)}\chi_{22}^{\left(2\right)})\gamma_{2}^{\left(2\right)}e^{s\gamma_{2}^{\left(2\right)}d_{2}}(\chi_{21}^{\left(1\right)}-\chi_{22}^{\left(1\right)}),$$

$$B_{1}^{*}=(c_{44}^{\left(2\right)}\chi_{12}^{\left(2\right)}+e_{24}^{\left(2\right)}\chi_{22}^{\left(2\right)})\gamma_{2}^{\left(2\right)}e^{s\gamma_{2}^{\left(2\right)}d_{2}}(\chi_{12}^{\left(1\right)}-\chi_{11}^{\left(1\right)}),$$

$$A_{2}^{*}=(c_{44}^{\left(2\right)}\chi_{11}^{\left(2\right)}+e_{24}^{\left(2\right)}\chi_{21}^{\left(2\right)})\gamma_{1}^{\left(2\right)}e^{s\gamma_{1}^{\left(2\right)}d_{2}}(\chi_{22}^{\left(1\right)}-\chi_{21}^{\left(1\right)}),$$

$$B_{2}^{*}=(c_{44}^{\left(2\right)}\chi_{11}^{\left(2\right)}+e_{24}^{\left(2\right)}\chi_{21}^{\left(2\right)})\gamma_{1}^{\left(2\right)}e^{s\gamma_{1}^{\left(2\right)}d_{2}}(\chi_{11}^{\left(1\right)}-\chi_{12}^{\left(1\right)}),$$

$$\begin{array}{c} C_{1}^{*}=((c_{44}^{\left(1\right)}\chi_{11}^{\left(1\right)}+e_{24}^{\left(1\right)}\chi_{21}^{\left(1\right)})\gamma_{1}^{\left(1\right)}-(c_{44}^{\left(1\right)}\chi_{12}^{\left(1\right)}+e_{24}^{\left(1\right)}\chi_{22}^{\left(1\right)})\gamma_{2}^{\left(1\right)})((c_{44}^{\left(2\right)}\chi_{12}^{\left(2\right)}+e_{24}^{\left(2\right)}\chi_{22}^{\left(2\right)})\gamma_{2}^{\left(2\right)}\chi_{21}^{\left(2\right)}\\ -(c_{44}^{\left(2\right)}\chi_{11}^{\left(2\right)}+e_{24}^{\left(2\right)}\chi_{21}^{\left(2\right)})\gamma_{1}^{\left(2\right)}\chi_{22}^{\left(2\right)}) \end{array},$$

$$\begin{array}{c} C_{2}^{*}=((c_{44}^{\left(1\right)}\chi_{11}^{\left(1\right)}+e_{24}^{\left(1\right)}\chi_{21}^{\left(1\right)})\gamma_{1}^{\left(1\right)}-(c_{44}^{\left(1\right)}\chi_{12}^{\left(1\right)}+e_{24}^{\left(1\right)}\chi_{22}^{\left(1\right)})\gamma_{2}^{\left(1\right)})((c_{44}^{\left(2\right)}\chi_{11}^{\left(2\right)}+e_{24}^{\left(2\right)}\chi_{21}^{\left(2\right)})\gamma_{1}^{\left(2\right)}\chi_{12}^{\left(2\right)}\\ -(c_{44}^{\left(2\right)}\chi_{12}^{\left(2\right)}+e_{24}^{\left(2\right)}\chi_{22}^{\left(2\right)})\gamma_{2}^{\left(2\right)}\chi_{11}^{\left(2\right)}) \end{array},$$

$$\begin{array}{c} C_{3}^{*}=((e_{15}^{\left(1\right)}\chi_{11}^{\left(1\right)}-\varepsilon_{22}^{\left(1\right)}\chi_{21}^{\left(1\right)})(c_{44}^{\left(2\right)}\chi_{12}^{\left(2\right)}+e_{24}^{\left(2\right)}\chi_{22}^{\left(2\right)})-(e_{15}^{\left(1\right)}\chi_{12}^{\left(1\right)}-\varepsilon_{22}^{\left(1\right)}\chi_{22}^{\left(1\right)})(c_{44}^{\left(2\right)}\chi_{11}^{\left(2\right)}\\ +e_{24}^{\left(2\right)}\chi_{21}^{\left(2\right)}))\gamma_{1}^{\left(1\right)}\gamma_{2}^{\left(2\right)}(\chi_{21}^{\left(1\right)}-\chi_{22}^{\left(1\right)}) \end{array},$$

$$\begin{array}{c} C_{4}^{*}=((e_{15}^{\left(1\right)}\chi_{11}^{\left(1\right)}-\varepsilon_{22}^{\left(1\right)}\chi_{21}^{\left(1\right)})(c_{44}^{\left(2\right)}\chi_{12}^{\left(2\right)}+e_{24}^{\left(2\right)}\chi_{22}^{\left(2\right)})-(e_{15}^{\left(1\right)}\chi_{12}^{\left(1\right)}-\varepsilon_{22}^{\left(1\right)}\chi_{22}^{\left(1\right)})(c_{44}^{\left(2\right)}\chi_{11}^{\left(2\right)}\\ +e_{24}^{\left(2\right)}\chi_{21}^{\left(2\right)}))\gamma_{1}^{\left(1\right)}\gamma_{2}^{\left(2\right)}(\chi_{12}^{\left(1\right)}-\chi_{11}^{\left(1\right)}) \end{array},$$

$$\begin{array}{c} B^{*}=(c_{44}^{\left(2\right)}\chi_{11}^{\left(2\right)}+e_{24}^{\left(2\right)}\chi_{21}^{\left(2\right)})\gamma_{1}^{\left(2\right)}(-\chi_{11}^{\left(1\right)}\chi_{22}^{\left(2\right)}+\chi_{12}^{\left(2\right)}\chi_{21}^{\left(1\right)}+\chi_{12}^{\left(1\right)}\chi_{22}^{\left(2\right)}-\chi_{12}^{\left(2\right)}\chi_{22}^{\left(1\right)})\\ +(c_{44}^{\left(2\right)}\chi_{12}^{\left(2\right)}+e_{24}^{\left(2\right)}\chi_{22}^{\left(2\right)})\gamma_{2}^{\left(2\right)}(-\chi_{11}^{\left(2\right)}\chi_{21}^{\left(1\right)}+\chi_{11}^{\left(1\right)}\chi_{21}^{\left(2\right)}-\chi_{12}^{\left(1\right)}\chi_{21}^{\left(2\right)}+\chi_{11}^{\left(2\right)}\chi_{22}^{\left(1\right)}) \end{array}.$$
